# Effect of different courses and durations of invasive mechanical ventilation on respiratory outcomes in very low birth weight infants

**DOI:** 10.1038/s41598-023-46456-7

**Published:** 2023-11-03

**Authors:** Yang Yang, Xin-yue Gu, Zhen-lang Lin, Shu-lin Pan, Jian-hua Sun, Yun Cao, Shoo K. Lee, Jian-hui Wang, Rui Cheng, the CHNN investigators, Shoo K. Lee, Chao Chen, Lizhong Du, Wenhao Zhou, Yun Cao, Xiuyong Chen, Huayan Zhang, Xiuying Tian, Yong Ji, Zhankui Li, Bing Yi, Chuanzhong Yang, Ling Liu, Jianhua Fu, Sannan Wang, Dongmei Chen, Changyi Yang, Xirong Gang, Rui Cheng, Hui Wu, Ruobing Shan, Shuping Han, Xinnian Pan, Yiheng Dai, Lili Wang, Gang Qiu, Mingxia Li, Hong Jiang, Wenqing Kang, Yuan Shi, Jiangqin Liu, Qin Zhou, Xing Feng, Jiahua Pan, Long Li, Hongping Xia, Zhenlang Lin, Pingyang Chen, Qin Zhang, Falin Xu, Ling Yang, Xinzhu Lin, Xiaoying Li, Li Ma, Deyi Zhuang, Jianhua Sun, Kun Liang, Yinping Qiu, Xiaoyun Zhong, Jinxing Feng, Liping Chen, Mingyan Hei, Wenbin Dong, Huiwen Huang, Zhaoqing Yin, Li Li, Jie Yang, Liyan Zhang, Hongxia Song, Huaiyan Wang, Yanhong Li, Jimei Wang, Hongying Mi, Dong Li, Linkong Zeng, Hongdan Zhu, Qianshen Zhang, Guofang Ding, Yan Gao, Hongyun Wang, Zhen Wang, Hong Zhen, Xiaolu Ma, Fang Wu, Joseph Ting

**Affiliations:** 1 Department of Neonatology, Children’s Hospital of Nanjing Medical University, Nanjing, China; 2 NHC Key Laboratory of Neonatal Diseases (Fudan University), Children’s Hospital of Fudan University, Shanghai, China; 3 Department of Neonatology, Wenzhou Medical College Affiliated Yuying Children’s Hospital, Wenzhou, China; 4 Department of Neonatology, Shanghai Children’s Medical Center Affiliated with the School of Medicine of Shanghai Jiaotong University, Shanghai, China; 5 Department of Neonatology, Children’s Hospital of Fudan University, Shanghai, China; 6 Department of Pediatrics, Maternal-Infant Care Research Centre, Mount Sinai Hospital, Toronto, ON Canada; 7 Department of Pediatrics, University of Toronto, Toronto, ON Canada; 8 Department of Obstetrics and Gynecology, Dalla Lana School of Public Health, University of Toronto, Toronto, ON Canada; 9 Department of Neonatology, Children’s Hospital of Chongqing Medical University, Chongqing, China; 10 Mount Sinai Hospital, University of Toronto, Toronto, Canada; 11 Children’s Hospital of Fudan University, Shanghai, China; 12 Children’s Hospital of Zhejiang University School of Medicine, Hangzhou, China; 13 Site Principle Investigators of the Chinese Neonatal Network: Children’s Hospital of Fudan University, Shanghai, China; 14 The Third Affiliated Hospital of Zhengzhou University, Zhengzhou, China; 15 Guangzhou Women and Children’s Medical Center, Guangzhou, China; 16 Tianjin Obstetrics and Gynecology Hospital, Tianjin, China; 17 Children’s Hospital of Shanxi, Taiyuan, China; 18 Northwest Women’s and Children’s Hospital, Xi’an, China; 19 Gansu Provincial Maternity and Child Care Hospital, Lanzhou, China; 20 Shenzhen Maternity and Child Health Care Hospital, Shenzhen, China; 21 Guizhou Women and Children’s Hospital, Guiyang, China; 22 Shengjing Hospital of China Medical University, Shenyang, China; 23 Suzhou Municipal Hospital affiliated to Nanjing Medical University, Suzhou, China; 24 Quanzhou Women and Children’s Hospital, Guilin, China; 25 Fujian Women and Children’s Medical Center, Fuzhou, China; 26 Hunan Children’s Hospital, Changsha, China; 27 Children’s Hospital of Nanjing Medical University, Nanjing, China; 28 The First Bethune Hospital of Jilin University, Changchun, China; 29 Qingdao Women and Children’s Hospital, Qingdao, China; 30 Nanjing Maternity and Child Health Care Hospital, Nanjing, China; 31 Women and Children’s Hospital of Guangxi Zhuang Autonomous Region, Nanning, China; 32 Foshan Women and Children’s Hospital, Foshan, China; 33 The First Affiliated Hospital of Anhui Medical University, Hefei, China; 34 Children’s Hospital of Shanghai, Shanghai, China; 35 The First Affiliated Hospital of Xinjiang Medical University, Ürümqi, China; 36 The Affiliated Hospital of Qingdao University, Shinan, China; 37 Henan Children’s Hospital, Zhengzhou, China; 38 Children’s Hospital of Chongqing Medical University, Chongqing, China; 39 Shanghai First Maternity and Infant Hospital, Shanghai, China; 40 Wuxi Maternity and Child Healthcare Hospital, Wuxi, China; 41 Children’s Hospital of Soochow University, Suzhou, China; 42 Anhui Provincial Hospital, Hefei, China; 43 People’s Hospital of Xinjiang Uygur Autonomous Region, Ürümqi, China; 44 Xinhua Hospital Affiliated to Shanghai Jiaotong University School of Medicine, Shanghai, China; 45 Yuying Children’s Hospital Affiliated to Wenzhou Medical University, Wenzhou, China; 46 The Second XiangYa Hospital of Central South University, Changsha, China; 47 Shaanxi Provincial People’s Hospital, Xi’an, China; 48 The First Affiliated Hospital of Zhengzhou University, Zhengzhou, China; 49 Hainan Women and Children’s Hospital, HaiKou, China; 50 Xiamen Maternity and Child Health Care Hospital, Xiamen, China; 51 Qilu Children’s Hospital of Shandong University, Jinan, China; 52 Hebei Children’s Hospital, Shijiazhuang, China; 53 Xiamen Children’s Hospital, Xiamen, China; 54 Shanghai Children’s Medical Center Affiliated to Shanghai Jiaotong University School of Medicine, Shanghai, China; 55 First Affiliated Hospital of Kunming Medical University, Kunming, China; 56 General Hospital of Ningxia Medical University, Yinchuan, China; 57 Chongqing Health Care Center for Women and Children, Chongqing, China; 58 Shenzhen Children’s Hospital, Shenzhen, China; 59 Jiangxi Provincial Children’s Hospital, Nanchang, China; 60 Beijing Children’s Hospital of Capital Medical University, Beijing, China; 61 The Affiliated Hospital of Southwest Medical University, Luzhou, China; 62 Zhuhai Center for Maternal and Child Health Care, Zhuhai, China; 63 Dehong People’s Hospital of Yunnan Province, Jianchuan, China; 64 Children’s Hospital Affiliated to Capital Institute of Pediatrics, Beijing, China; 65 Guangdong Women and Children’s Hospital, Guangzhou, China; 66 Fuzhou Children’s Hospital of Fujian Province, Fuzhou, China; 67 First Affiliated Hospital of Xian Jiaotong University, Xi’an, China; 68 Changzhou Maternal and Children Health Care Hospital, Changzhou, China; 69 Ningbo Women and Children Hospital, Ningbo, China; 70 Obstetrics and Gynecology Hospital of Fudan University, Shanghai, China; 71 The First People’s Hospital of Yunnan Province, Kunming, China; 72 Dalian Municipal Women and Children’s Medical Center, Dalian, China; 73 Wuhan Children’s Hospital, Wuhan, China; 74 Maternal and Children Hospital of Shaoxing, Shaoxing, China; 75 Shenzhen Hospital of Hongkong University, Shenzhen, China; 76 Peking Union Medical College Hospital, Beijing, China; 77 Lianyungang Maternal and Children Health Hospital, Lianyungang, China; 78 Inner Mongolia Maternal and Child Health Care Hospital, Hulunbuir, China; 79 Qinghai Women and Children Hospital, Xining, China; 80 Anhui Children’s Hospital, Hefei, China; 81 Children’s Hospital of Zhejiang University, Hangzhou, China; 82 Shanghai General Hospital, Shanghai, China; 83 University of Alberta, Edmonton, Canada

**Keywords:** Respiratory tract diseases, Respiratory distress syndrome

## Abstract

This multicenter retrospective study was conducted to explore the effects of different courses and durations of invasive mechanical ventilation (MV) on the respiratory outcomes of very low birth weight infants (VLBWI) in China. The population for this study consisted of infants with birth weight less than 1500 g needing at least 1 course of invasive MV and admitted to the neonatal intensive care units affiliated with the Chinese Neonatal Network within 6 h of life from January 1st, 2019 to December 31st, 2020. Univariate and multivariate logistic regression analyses were performed to evaluate associations between invasive MV and respiratory outcomes. Adjusted odds ratios (ORs) were computed with the effects of potential confounders. (1) Among the 3183 VLBWs with a history of at least one course of invasive MV, 3155 (99.1%) met inclusion criteria and were assessed for the primary outcome. Most infants received one course (76.8%) and a shorter duration of invasive MV (62.16% with ventilation for 7 days or less). (2) In terms of the incidence of all bronchopulmonary dysplasia (BPD) (mild, moderate, and severe BPD), there were no significant differences between different invasive MV courses [For 2 courses, adjusted OR = 1.11 (0.88, 1.39); For 3 courses or more, adjusted OR = 1.07 (0.72, 1.60)]. But, with the duration of invasive MV prolonging, the OR of BPD increased [8–21 days, adjusted OR = 1.98 (1.59, 2.45); 22–35 days, adjusted OR = 4.37 (3.17, 6.03); ≥ 36 days, adjusted OR = 18.44 (10.98, 30.99)]. Concerning severe BPD, the OR increased not only with the course of invasive MV but also with the duration of invasive MV [For 2 courses, adjusted OR = 2.17 (1.07, 4.40); For 3 courses or more, adjusted OR = 2.59 (1.02, 6.61). 8–21 days, adjusted OR = 8.42 (3.22, 22.01); 22–35 days, adjusted OR = 27.82 (9.08, 85.22); ≥ 36 days, adjusted OR = 616.45 (195.79, > 999.999)]. (3) When the interaction effect between invasive MV duration and invasive MV course was considered, it was found that there were no interactive effects in BPD and severe BPD. Greater than or equal to three courses would increase the chance of severe BPD, death, and the requirement of home oxygen therapy. Compared with distinct courses of invasive MV, a longer duration of invasive MV (> 7 days) has a greater effect on the risk of BPD, severe BPD, death, and the requirement of home oxygen therapy.

## Introduction

In mainland China, premature births are growing in frequency with the improved assisted reproductive technology and the increasing pregnancy rate at advanced maternal age^1^. However, because of immature lung development, many premature babies develop respiratory distress syndrome (RDS), apnea, pulmonary hemorrhage, and other respiratory complications, which result in the need for intubation and subsequent invasive mechanical ventilation (MV)^2–4^. Unfortunately, invasive MV is closely related to short-term and long-term complications such as atelectasis, air leak syndrome, ventilator-associated pneumonia, chronic lung disease, and neurodevelopmental disorders^5,6^. Therefore, an important issue faced by neonatologists is how to decrease the incidence and severity of respiratory diseases, thus reducing the need for invasive MV.

Existing studies have shown that early removal from invasive MV can lower the risk of airway injury, bronchopulmonary dysplasia (BPD), and other complications^7,8^. However, a considerable number of extremely preterm infants [gestational age (GA) < 28 weeks] still experience extubation failure and long-term invasive MV. 15–20% of these infants require three or more courses of invasive ventilation^9^. In very low birth weight infants (VLBWIs), the association between invasive MV and poor prognosis, including respiratory diseases, has not been fully clarified. Jensen et al. retrospectively analyzed the data from the US neonatal collaboration network and found that among all extremely low birth weight infants (ELBWIs), the risk of BPD was significantly higher in the multiple invasive MV courses group. However, concerning the surviving ELBWIs, the risk of BPD was not significantly different in distinct courses of invasive MV. In addition, they found that a longer duration of invasive MV was associated with a higher odds ratio (OR) of oxygen inhalation at discharge^10^. As far as mainland China is concerned, there has been no multicenter report on the above issues in VLBWIs. Given this situation, this paper conducted a retrospective multicenter study on VLBWIs and analyzed the effects of different courses as well as different durations of invasive MV on the respiratory outcomes of Chinese VLBWIs.

## Methods

### The Chinese neonatal network and database

The Chinese Neonatal Network (CHNN) is a national network of Chinese tertiary neonatal intensive care units (NICUs) with the primary goal of conducting high-quality collaborative research dedicated to the improvement of neonatal-perinatal health in China. Hospitals enrolled in CHNN are required to be tertiary referral hospitals with large neonatal services and recognized expertise in caring for high-risk neonates. In 2019, 57 NICUs in 25 provinces and municipalities contributed to the CHNN, which prospectively collects standardized data on demographics, outcomes, selected clinical practices, and resource use on all infants born at 24.0–31.9 weeks GA or with a birth weight < 1500 g and admitted to participating hospitals.

This project was approved by the CHNN Executive Committee and the Research Ethics Board at the Children’s Hospital of Fudan University, Shanghai, China^11^. All methods were performed by the relevant guidelines and regulations.

### Study population

The population for this study consisted of infants needing at least 1 course of invasive MV with birth weight less than 1500 g and GA less than 32 weeks. The subjects were admitted to the tertiary NICUs in the CHNN within 6 h of life from January 1st, 2019 to December 31st, 2020. We excluded any infants with major congenital anomalies.

### Outcome

Primary outcome: Based on the criteria of the National Institute of Child Health and Human Development 2001, BPD is defined and graded as respiratory support at 36 weeks’ postmenstrual age (PMA) or discharge (whichever is earlier). Moderate BPD is defined as the persistent need for oxygen [fraction of inspiration O_2_ (FiO_2_) < 30%] at 36 weeks’ PMA. Severe BPD is defined as the persistent need for oxygen (FiO_2_ ≥ 30%) and/or ventilatory support (invasive or non-invasive MV) at 36 weeks’ PMA^12^.

Secondary outcomes: (1) mortality; (2) incidence of patent ductus arteriosus (PDA)^13^; (3) incidence of necrotizing enterocolitis (NEC) (Bell stage ≥ II)^14^; (4) incidence of severe retinopathy of prematurity (ROP) (stage ≥ 3)^15^); (5) incidence of periventricular leukomalacia (PVL)^16^; (6) incidence of cultural-proven sepsis; (7) length of hospital stay; (8) requirement of home oxygen therapy.

### Exposure variable

Invasive MV means mechanical ventilation by endotracheal tube. The number of courses and durations of invasive MV were set as the exposure variable. Consequently, the study was first divided into three groups according to different courses: (1) 1 course of ventilation; (2) 2 courses of ventilation; (3) 3 courses of ventilation or more. Moreover, the VLBWIs were divided into four groups according to different durations of invasive MV: (1) ≤ 7d; (2) 8–21d; (3) 22–35d; (4) ≥ 36d. Distinct invasive MV courses were identified if separated by more than 24 h without MV. Cases where there was an interval of less than 24 h were still considered as one course of invasive ventilation.

### Statistical analysis

Descriptive statistical methods were used to summarize the study population. The characteristics and outcomes were compared for multiple comparisons using the χ^2^ test for categorical variables, and the Student *t*-test or Wilcoxon rank-sum test, as appropriate, for continuous variables. We used a multivariate logistic regression model to adjust for potential confounders that were identified in the univariate analysis or based on clinical experience and previous reference^10^. This includes GA, birth weight, sex, small for gestational age (SGA), 5-min Apgar score, treatment with surfactant, treatment with dexamethasone (DART), PDA, NEC, cultural-proven sepsis during hospitalization, invasive MV duration, invasive MV course. To explore the effect of courses and duration of ventilation on BPD, we used the above model for determining adjusted ORs. In addition, the interaction effect between invasive MV duration and invasive MV course was also considered for further analysis.

Besides, we conducted subgroup analysis stratified by GA groups (GA ≤ 26 weeks, 26w < GA ≤ 28weeks, and GA ≥ 28 weeks) using the same multivariate logistic regression model (Supplements- supplementary tables). Data management and all statistical analysis were performed using SAS version 9.4 (SAS Institute, Inc., Cary, NC, USA). The two-sided *p*-value of 0.05 was used to determine statistical significance.

### Statement of ethics

This study was approved by the Ethics Review Board of Children’s Hospital of Fudan University, Approval No. 2018296. The requirement for informed consent was waived by the Ethics Review Board of the Children’s Hospital of Fudan University.

## Results

### Characteristics of the study participants

Among the 3183 VLBWs with a history of at least one course of invasive MV, 28 infants with major congenital anomalies were excluded. 3155 (99.1%) were finally included and assessed for the primary outcome. Demographic characteristics are given in Table 1. GA, birth weight, male, DART treatment, surfactant treatment, and invasive MV duration exhibited significant differences between distinct courses of invasive MV. Meanwhile, GA, birth weight, male, SGA, 5 min Apgar, DART treatment, surfactant treatment, and invasive MV courses showed statistical differences among groups with different invasive MV durations. Most infants received one course [76.8%] and a shorter duration of invasive MV [62.16% with ventilation for 7 days or less]. (Table 1).

**Table 1:** Characteristics of included infants based on mechanical ventilation courses and duration.

Demographics	Number of mechanical ventilation course				Duration of mechanical ventilation				
	1 course (N = 2422)	2 courses (N = 578)	≥ 3 courses (N = 155)	*p*-value	≤ 7d (N = 1961)	8-21d (N = 775)	22-35d (N = 266)	≥ 36d (N = 153)	*p*-value
Maternal characteristics									
Maternal age, mean (SD), years	31.1 (4.9)	31.3 (4.9)	32.0 (4.4)	0.05	31.0 (5.0)	31.4 (4.9)	31.3 (4.7)	31.07 (4.3)	0.11
Maternal diabetes, n/N (%)	459/2406 (19.1)	119/575 (20.7)	27/153 (17.7)	0.59	370/1950 (19.0)	151/770 (19.6)	55/262 (21.0)	29/152 (19.1)	0.88
Maternal hypertension, n/N (%)	627/2407 (26.1)	135/575 (23.5)	39/154 (25.3)	0.45	516/1951 (26.5)	204/771 (26.5)	55/263 (20.9)	26/151 (17.2)	0.02
Chorioamnionitis, n/N (%)	391/1883 (20.8)	107/446 (24.0)	33/130 (25.4)	0.18	318/1562 (20.4)	143/585 (24.4)	44/201 (21.9)	26/111 (23.4)	0.22
PROM > 24 h, n/N (%)	490/2299 (21.3)	110/542 (20.3)	36/151 (23.8)	0.64	413/1876 (22.0)	146/730 (20.0)	49/240 (20.4)	28/146 (19.2)	0.61
Perinatal glucocorticoid, n/N (%)	1820/2316 (78.6)	436/556 (78.4)	118/148 (79.7)	0.94	1498/1882 (79.6)	580/741 (78.3)	191/252 (75.8)	105/145 (72.4)	0.13
Infants’ characteristics									
Gestational age, mean (SD), weeks	29.2 (1.8)	28.4 (2.0)	27.9 (1.8)	< .01	29.5 (1.75)	28.7 (1.7)	27.7 (1.9)	26.8 (1.8)	< .01
Birth weight, median (IQR), grams	1190 (1020–1340)	1060 (905–1250)	970 (840–1170)	< .01	1200 (1050–1350)	1100 (965–1270)	997 (850–1150)	850 (740–1030)	< .01
Male, n/N (%)	1,299/2422 (53.6)	344/578 (59.5)	100/155 (64.5)	< .01	1032/1960 (52.7)	447/775 (57.7)	181/266 (68.1)	83/153 (54.3)	< .01
SGA, n/N (%)	350/2418 (14.5)	70/573 (12.2)	19/154 (12.3)	0.31	303/1961 (15.5)	94/775 (12.1)	27/262 (10.3)	15/147 (10.2)	0.02
5 min Apgar score, median (IQR)	9 (8–9)	9 (8–9)	9 (8–9)	0.07	9 (8–9)	9 (8–9)	8 (7–9)	8 (7–9)	< .01
DART treatment, n/N (%)	361/2422 (14.9)	148/578 (25.6)	44/155 (28.4)	< .01	185/1961 (9.4)	172/775 (22.2)	107/266 (40.2)	89/153 (58.2)	< .01
Surfactant treatment, n/N (%)	1836/2422 (75.8)	487/578 (84.3)	135/155 (87.1)	< .01	1450/1961 (73.9)	636/775 (82.1)	241/266 (90.6)	131/153 (85.6)	< .01
Mechanical ventilation duration, median (IQR), days	4 (2–8)	13 (8–22)	21 (14–30)	< .01	3 (2–5)	12 (9–15)	27 (24–31)	46 (39–57)	< .01
Mechanical ventilation courses, median (IQR)	1 (1–1))	2 (2–2)	3 (3–3)	< .01	1 (1–1)	1 (1–2)	2 (1–2)	2 (1–2)	< .01

*SD* standard deviation, *IQR* interquartile range, *PROM* premature rupture of membranes, *SGA* small for gestational age, *DART* dexamethasone: a randomized trial.

### Outcomes

It was found that the requirement of home oxygen therapy, length of hospital stay as well as incidences of BPD, severe BPD, PDA, NEC (Bell stage ≥ II), sepsis, severe ROP (stage ≥ 3), and death showed increasing trends with the ventilation courses growing. Similar results could be found among different invasive MV durations. (Table 2).

**Table 2:** Rates of outcomes before NICU discharge based on mechanical ventilation courses and duration.

Variable	Number of mechanical ventilation courses				Duration of mechanical ventilation				
	1 course	2 courses	≥ 3 courses	*p*-value	≤ 7d	8-21d	22-35d	≥ 36d	*p*-value
BPD, n/N (%)	613/2422 (25.3)	239/578 (41.4)	81/155 (52.3)	< .01	385/1961 (19.6)	270/775 (34.8)	147/266 (55.3)	131/153 (85.6)	< .01
Severe BPD, n/N (%)	38/2422 (1.6)	27/578 (4.7)	16/155 (10.3)	< .01	10/1961 (0.5)	17/775 (2.2)	11/266 (4.1)	43/153 (28.1)	< .01
Death, n/N (%)	67/2422 (2.8)	44/578 (7.6)	24/155 (15.5)	< .01	42/1961 (2.1)	35/775 (4.5)	41/266 (15.4)	17/153 (11.1)	< .01
PDA, n/N (%)	1324/2403 (55.1)	359/578 (62.1)	103/154 (66.9)	< .01	958/1944 (49.3)	504/774 (65.1)	198/264 (75.0)	126/153 (82.4)	< .01
NEC (grade ≥ II), n/N (%)	148/2422 (6.1)	76/578 (13.2)	29/155 (18.7)	< .01	143/1961 (7.3)	70/775 (9.0)	20/266 (7.5)	20/153 (13.1)	0.05
Sepsis (confirmed by blood culture)	288/2422 (11.9)	125/578 (21.6)	50/155 (32.3)	< .01	233/1961 (11.9)	142/775 (18.3)	57/266 (21.4)	31/153 (20.3)	< .01
Severe ROP, n/N (%)	102/2272 (4.5)	56/532 (10.5)	24/144 (16.7)	< .01	54/1846 (2.9)	63/726 (8.7)	29/231 (12.6)	36/145 (24.8)	< .01
PVL, n/N (%)	166/286 (7.3)	54/541 (10.0)	15/150 (10.0)	0.07	106/1843 (5.8)	81/734 (11.0)	33/253 (13.0)	15/147 (10.2)	< .01
Requirement of home oxygen therapy, n/N (%)	352/2400 (14.7)	139/562 (24.7)	55/147 (37.4)	< .01	223/1945 (11.5)	166/768 (21.6)	95/251 (37.9)	62/145 (42.8)	< .01
Length of hospital stay, median (IQR), days	57 (45–70)	71 (56–87)	78 (62–97)	< .01	55 (44–67)	66 (53–81)	75 (58–96)	98 (80–125)	< .01

*BPD* bronchopulmonary dysplasia, *PDA* patent ductus arteriosus, *NEC* necrotizing enterocolitis, *ROP* retinopathy of prematurity, *PVL* periventricular leukomalacia, *IQR* interquartile range.

### Adjusted ORs for respiratory outcomes

As far as BPD is concerned, there were no significant differences between different ventilation courses. Compared with that, the OR of BPD increased with the duration of invasive MV prolonging. With regard to severe BPD and death, the ORs evidently increased not only with the course of invasive MV but also with the duration of invasive MV. In comparison with the course of MV, the ventilation duration had a greater impact on the need for home oxygen therapy and the requirement of MV at discharge. (Table 3).

**Table 3:** Adjusted odds ratios for respiratory outcomes before NICU discharge among very low birth weight infants received different courses and duration of mechanical ventilation.

Outcome	Number of mechanical ventilation courses^a^			Duration of mechanical ventilation ^b^			
	1 course	2 courses	≥ 3 courses	≤ 7d	8-21d	22-35d	≥ 36d
BPD	Reference	1.11 (0.88, 1.39)	1.07 (0.72, 1.60)	Reference	1.98 (1.59, 2.45)	4.37 (3.17, 6.03)	18.44 (10.98, 30.99)
Severe BPD	Reference	2.17 (1.07, 4.40)	2.59 (1.02, 6.61)	Reference	8.42 (3.22, 22.01)	27.82 (9.08, 85.22)	616.45 (195.79, > 999.999)
Death	Reference	1.80 (1.17, 2.77)	2.52 (1.41, 4.51)	Reference	1.70 (1.01, 2.84)	6.31 (3.53, 11.26)	3.78 (1.79, 8.00)
Requirement of home oxygen therapy	Reference	1.20 (0.94, 1.54)	1.62 (1.07, 2.43)	Reference	1.82 (1.42, 2.34)	3.75 (2.65, 5.30)	4.24 (2.74, 6.57)
Discharged with requirement of non-invasive/invasive mechanical ventilation	Reference	1.47 (0.96, 2.25)	1.89 (1.04, 3.42)	Reference	1.70 (1.03, 2.80)	5.59 (3.14, 9.95)	3.00 (1.45, 6.23)

^a^Adjusted for gestational age, birth weight, sex, small for gestational age, 5-min Apgar score, treatment with surfactant, DART treatment, PDA, NEC, sepsis, and mechanical ventilation duration (analyzed as grouping variable).

^b^Adjusted for gestational age, birth weight, sex, small for gestational age, 5-min Apgar score, treatment with surfactant, DART treatment, PDA, NEC, sepsis, and mechanical ventilation courses (analyzed as grouping variable).

In addition, when the interaction effect was considered, it was found that death and requirement of home oxygen therapy showed significant interactive effect with invasive MV duration and invasive MV course. Compared with the above outcomes, there were no interactive effects in BPD, severe BPD, and the requirement of non-invasive/invasive MV at discharge. (Table 4).

**Table 4:** Adjusted odds ratios for respiratory outcomes before NICU discharge among very low birth weight infants received different courses and duration of mechanical ventilation when considering the interaction effect.

Outcome	Number of mechanical ventilation courses			Duration of mechanical ventilation				Interaction term#
	1 course	2 courses	≥ 3 courses	≤ 7d	8-21d	22-35d	≥ 36d	*p*-value
BPD	Reference	1.45 (0.96, 2.19)	0.53 (0.06, 4.62)	Reference	2.11 (1.64, 2.72)	4.84 (3.17, 7.39)	15.27 (8.01, 29.12)	0.5864
Severe BPD	Reference	10.27 (2.19, 48.22)	N/A*	Reference	8.29 (1.98, 34.66)	67.85 (16.51, 278.86)	982.79 (249.24, > 999.999)	0.0838
Death	Reference	1.59 (0.64, 3.98)	11.08 (1.84, 66.57)	Reference	0.97 (0.42, 2.24)	8.21 (3.95, 17.09)	7.07 (2.98, 16.78)	0.0172
Requirement of home oxygen therapy	Reference	1.07 (0.63, 1.81)	38.74 (4.42, 339.64)	Reference	1.76 (1.30, 2.38)	4.22 (2.70, 6.58)	5.39 (3.12, 9.34)	0.0382
Discharged with requirement of non-invasive/invasive mechanical ventilation	Reference	0.94 (0.32, 2.77)	13.12 (1.78, 96.40)	Reference	1.23 (0.61, 2.50)	7.55 (3.77, 15.12)	3.99 (1.65, 9.65)	0.1438

Adjusted for gestational age, birth weight, sex, small for gestational age, 5-min Apgar score, treatment with surfactant, DART treatment, PDA, NEC, sepsis, mechanical ventilation duration (analyzed as grouping variable), and mechanical ventilation courses (analyzed as grouping variable). *N/A: Not Applicable. No valid statistical result was found because of the limited number of valid events. # Interaction term here refers to invasive MV duration*invasive MV course.

## Discussion

Due to immature development of lung and cerebral respiratory center, it is not uncommon for VLBWIs requiring reintubation after extubation failure. In mainland China, although there have been no large multicenter studies, according to recent cohort studies from other parts of the world, nearly 50% of VLBWIs and 60–70% of ELBWIs need at least two or more courses of invasive MV during hospitalization^17,18^. Shalish et al. found that reintubation was associated with lower gestational age/birth weight and greater morbidities compared with infants never reintubated in 216 newborns whose birth weight ≤ 1250 g^19^. Yossef et al. included 210 patients born at < 27 weeks from 2005 to 2011. They further found that long-term intermittent positive pressure ventilation patients were born earlier and had lower birth weight^20^. Markestad et al. performed a prospective observational study of 636 infants with a GA of 22–27 weeks or a birth weight of 500–999 g. For the survivors, days of invasive MV decreased from a median of 37 days to 3 days, and the proportion in need of oxygen at 36 weeks’ PMA decreased from 67 to 26% at 23 and 27 weeks’ GA, respectively^21^. The above evidence reflects the trend that more courses and longer duration of invasive MV are needed with decreasing GA and birth weight.

Because the course and duration of invasive MV are related to many potential reasons such as GA, birth weight, as well as sex. Besides the above known self-factors, the surfactant replacement and DART treatment are also closely associated with invasive MV. With the promotion of surfactant and dexamethasone therapies in the past several years, the application and duration of invasive MV have significantly been reduced and shortened. Our study found that the application of DART increases as the course and duration of invasive MV grow (Table 1). A similar growth trend can also be seen in surfactant administration (Table 1). This, to some extent, indicates that newborns who require multiple and long-term invasive MV have worse lung development and maturity. Univariate analysis of the outcomes firstly showed that multiple courses and longer duration of invasive MV are associated with the increased risks of BPD, severe BPD, PDA, death, sepsis, severe ROP, home oxygen therapy as well as longer hospital stay (Table 2). The above association has been partly confirmed by literature. For example, Tseng et al. found that invasive MV significantly increased the risk of ROP in a multicenter cohort study (n = 1,703,326, *p* < 0.0001)^22^. And, a persistent left-to-right shunt through PDA may impair pulmonary mechanics and is correlated with prolonged invasive MV needs^23^. In addition, Morrow et al. once performed a retrospective analysis of 660 newborns (GA ≤ 32 weeks) with BPD. The result showed that longer hospitalization was closely associated with invasive MV, supplemental oxygen need, etc^24^.

Through further multivariate analysis, we evaluated the effect of longer duration and multiple courses of invasive MV on respiratory outcomes. In terms of BPD and severe BPD, it was found that longer duration not only increased the incidence of BPD, but also the risks of severe BPD and home oxygen therapy (Tables 3 and 4). In fact, the incidences of BPD and severe BPD have been proven associated with the duration of invasive MV. Previous studies have explored the association between the duration of invasive MV and BPD. For example, a retrospective whole-population study including all infants with GA < 28 weeks from the UK (n = 11,806) found invasive MV duration had areas under the curve of 0.793 and 0.703 in predicting BPD and home oxygen therapy, respectively. MV for more than 8 days predicted BPD development with 71% sensitivity and 71% specificity^25^. A prospective multicenter cohort study included 6,538 infants born at 23–31 weeks of GA who were admitted to 47 NICUs in China from January 2018 to December 2021. It further revealed that the onset of moderate-to-severe BPD was significantly associated with the duration of initial invasive MV (adjusted OR 1.97; 95% CI 1.10–2.67)^26^. For severe BPD, a single-center, retrospective cohort study included infants born at 22.0–25.9 weeks during 2005–2009 (88 infants in the early era) and 2011–2015 (102 infants in the late era), which was before and after implementing guidelines recommending delayed extubation. The result showed that the incidence of severe BPD was significantly higher, and the duration of invasive MV was longer in the late era (after adopting delayed extubation)^27^.

Except for the duration, the number of courses of invasive MV is another possible factor associated with BPD. But the evidence so far is mixed. Subgroup analysis of two large randomized controlled trials showed that reintubation within 5 and 7 days after extubation is associated with an increased incidence of respiratory diseases including BPD^28,29^. But, a single-center retrospective study (n = 244) from Li et al. found that reintubation was not an independent risk factor for moderate-to-severe BPD (for reintubations occurring within 72 h after extubation, adjusted OR = 1.714, 95% CI 0.546–5.374; for reintubations occurring within 7 d after extubation, adjusted OR = 1.501, 95% CI 0.482–4.678)^30^. Shalish W et al. found that reintubation within observation windows ranging from 24 h to 3 weeks post-extubation was not associated with the increased ORs of BPD among survivors^19^. However, the above studies did not consider the interference of the ventilation duration as an important confounding factor. A multicenter retrospective study from Jensen et al. further showed that reintubation did not increase the risk of discharge on oxygen. More importantly, invasive MV for less than 3 courses did not increase the risk of BPD. Only in the population with more than 4 courses of invasive MV [MV duration 59 (41–87) days] did the risk of BPD increase compared with those infants with only 1 course [OR 1.44 (1.04–2.01)]^10^. Our study also found that multiple courses of invasive MV did not increase the risk of BPD (Tables 3 and 4). But, Jensen’s study did not consider the effect of different courses on the incidence of severe BPD. According to our findings, it turns out that distinct invasive MV courses would increase the risk of severe BPD, even considering the interaction effect between invasive MV duration and invasive MV course. As a matter of fact, the need for multiple courses of invasive MV generally means severe immature lung development, repeated pulmonary infection, or tracheomalacia in clinical practice. And those reasons are high-risk factors or important features of severe BPD. Furthermore, our results firstly manifested that after adjusting for confounding factors, multiple courses, especially greater than or equal to three courses would also increase the chance of death and requirement of home oxygen therapy. This could also be attributed to the elevated risk of severe BPD.

## Limitations

The main limitation of this study lies in the retrospective study design. And some factors such as the sample size of infants with severe BPD limit the reliability of the above results. It needs to be further studied by expanding the sample size in the future. In addition, extubation failure is an important and complicated issue. Extubation failure is common in extremely premature infants. However, many factors are related to extubation failure, such as lower birth weight, smaller GA, weaker spontaneous breath, different modes of MV, pneumonia, low vitality, etc. Since it is not the main objective of our research, the number of extubation failures and detailed time interval between two consecutive invasive MV were not further explored and included in this study.

## Conclusion

In conclusion, greater than or equal to three courses would increase the chance of severe BPD, death and requirement of home oxygen therapy. Compared with distinct courses of invasive MV, the longer duration of invasive MV (more than 7 days) has a greater effect on the risk of BPD, severe BPD, death, and requirement of home oxygen therapy.

### Supplementary Information

Supplementary Tables.

## Data Availability

All data generated or analyzed during this study are included in this article. Further inquiries can be directed to the corresponding author.
