# Supplementary material for: Effect of different courses and durations of invasive mechanical ventilation on respiratory outcomes in very low birth weight infants
Source: Sci Rep. 2023 Nov 3;13:18991. doi: 10.1038/s41598-023-46456-7 (PMC10624920; doi:10.1038/s41598-023-46456-7)
Supplement: Supplementary file 1 — Supplementary Tables. [file 41598_2023_46456_MOESM1_ESM.docx]

**Supplement table1.** Adjusted odds ratios for respiratory outcomes before NICU discharge among survived very low birth weight infants who received different courses and duration of mechanical ventilation

|  | **Number of mechanical ventilation courses ^a^** | | | **Duration of mechanical ventilation ^b^** | | | |
| --- | --- | --- | --- | --- | --- | --- | --- |
| **Outcome** | **1 course** | **2 courses** | **≥3 courses** | **≤7d** | **8-21d** | **22-35d** | **≥36d** |
| BPD | Reference | 1.00  (0.77, 1.29) | 0.87  (0.53, 1.40) | Reference | 2.04  (1.62, 2.57) | 6.23  (4.28, 9.07) | 30.75  (14.80, 63.89) |
| Severe BPD | Reference | 2.97  (1.22, 7.25) | 5.29  (1.59, 17.54) | Reference | 6.53  (2.03, 21.05) | 21.01  (5.62, 78.54) | 602.30  (146.95, >999.999) |
| Requirement of home oxygen therapy | Reference | 1.10  (0.80, 1.53) | 1.73  (1.02, 2.92) | Reference | 1.84  (1.34, 2.53) | 3.06  (1.94, 4.80) | 5.34  (3.09, 9.24) |

1. Adjusted for gestational age, birth weight, gender, small for gestational age, 5-min Apgar score, treatment with surfactant, DART treatment, PDA, NEC, sepsis, and mechanical ventilation duration (analyzed as continuous variable).
2. Adjusted for gestational age, birth weight, gender, small for gestational age, 5-min Apgar score, treatment with surfactant, DART treatment, PDA, NEC, sepsis, and mechanical ventilation courses (analyzed as continuous variable).

**Supplement table2.** Adjusted ORs for respiratory outcomes before NICU discharge among very low birth weight infants received different courses and duration of mechanical ventilation by gestational age

|  | **Number of mechanical ventilation courses ^a^** | | | **Duration of mechanical ventilation ^b^** | | | |
| --- | --- | --- | --- | --- | --- | --- | --- |
| **Outcome** | **1 course** | **2 courses** | **≥3 courses** | **≤7d** | **8-21d** | **22-35d** | **≥36d** |
| **GA>28w** (n=1617) |  |  |  |  |  |  |  |
| BPD | Reference | 0.91 (0.63, 1.32) | 1.06 (0.53, 2.13) | Reference | 1.96 (1.44, 2.66) | 7.68 (4.17, 14.13) | 52.41 (6.68, 411.14) |
| Severe BPD | Reference | 2.55 (0.99, 6.60) | 2.72 (0.70, 10.64) | Reference | 8.30 (2.96, 23.29) | 34.07 (9.17, 126.59) | 968.44 (192.08, >999.999) |
| Death | Reference | 2.32 (1.05, 5.17) | 3.47 (1.14, 10.54) | Reference | 2.28 (0.93, 5.60) | 7.64 (2.19, 26.63) | 38.85 (9.46, 159.53) |
| Discharged with requirement of home oxygen therapy | Reference | 0.99 (0.65, 1.51) | 1.21 (0.56, 2.63) | Reference | 2.10 (1.45, 3.03) | 2.75 (1.45, 5.23) | 18.15 (5.37, 61.34) |
| Discharged with requirement of non-invasive/invasive mechanical ventilation | Reference | 1.87 (0.83, 4.23) | 1.65 (0.44, 6.28) | Reference | 3.15 (1.30, 7.64) | 11.01 (3.01, 40.31) | 19.74 (3.77, 103.43) |
| **26w<GA≤28w** (n=1151) |  |  |  |  |  |  |  |
| BPD | Reference | 1.06 (0.73, 1.54) | 0.99 (0.53, 1.87) | Reference | 2.13 (1.49, 3.04) | 4.87 (2.89, 8.19) | 26.28 (11.39, 60.62) |
| Severe BPD | Reference | 0.82 (0.14, 4.84) | 1.68 (0.26, 10.89) | Reference | 3.32 (0.55, 19.90) | 11.67 (1.76, 77.28) | 218.74 (37.13, >999.999) |
| Death | Reference | 1.36 (0.69, 2.67) | 2.55 (1.07, 6.08) | Reference | 1.58 (0.74, 3.40) | 6.17 (2.62, 14.51) | 2.59 (0.81, 8.32) |
| Discharged with requirement of home oxygen therapy | Reference | 1.49 (1.01, 2.18) | 2.51 (1.36, 4.63) | Reference | 1.61 (1.09, 2.39) | 4.49 (2.62, 7.72) | 3.79 (1.92, 7.46) |
| Discharged with requirement of non-invasive/invasive mechanical ventilation | Reference | 1.32 (0.67, 2.59) | 1.73 (0.67, 4.48) | Reference | 1.48 (0.68, 3.20) | 5.89 (2.43, 14.23) | 3.00 (0.99, 9.07) |
| **GA≤26w** (n=387) |  |  |  |  |  |  |  |
| BPD | Reference | 1.44 (0.83, 2.48) | 1.11 (0.50, 2.46) | Reference | 1.38 (0.70, 2.73) | 1.95 (0.94, 4.05) | 9.45 (3.83, 23.33) |
| Severe BPD | Reference | 3.39 (0.44, 25.96) | 3.19 (0.31, 32.98) | Reference | 3.46 (0.23, 53.16) | 1.21 (0.04, 34.14) | 88.94 (6.16, >999.999) |
| Death | Reference | 1.33 (0.57, 3.09) | 1.41 (0.42, 4.71) | Reference | 0.49 (0.14, 1.72) | 2.03 (0.69, 5.96) | 0.66 (0.16, 2.78) |
| Discharged with requirement of home oxygen therapy | Reference | 0.79 (0.44, 1.42) | 1.04 (0.45, 2.36) | Reference | 1.10 (0.51, 2.36) | 2.18 (1.00, 4.75) | 2.38 (0.97, 5.83) |
| Discharged with requirement of non-invasive/invasive mechanical ventilation | Reference | 1.01 (0.46, 2.23) | 1.84 (0.67, 5.07) | Reference | 0.57 (0.19, 1.71) | 1.77 (0.64, 4.89) | 0.79 (0.22, 2.87) |

1. Adjusted for gestational age, birth weight, gender, small for gestational age, 5-min Apgar score, treatment with surfactant, DART treatment, PDA, NEC, sepsis, and mechanical ventilation duration (analyzed as continuous variable).
2. Adjusted for gestational age, birth weight, gender, small for gestational age, 5-min Apgar score, treatment with surfactant, DART treatment, PDA, NEC, sepsis, and mechanical ventilation courses (analyzed as continuous variable).

**Supplement table3.** Adjusted ORs for respiratory outcomes before NICU discharge among survived very low birth weight infants who received different courses and duration of mechanical ventilation by gestational age

|  | **Number of mechanical ventilation courses ^a^** | | | **Duration of mechanical ventilation ^b^** | | | |
| --- | --- | --- | --- | --- | --- | --- | --- |
| **Outcome** | **1 course** | **2 courses** | **≥3 courses** | **≤7d** | **8-21d** | **22-35d** | **≥36d** |
| **GA>28w** (n=1,431) |  |  |  |  |  |  |  |
| BPD | Reference | 0.88  (0.59, 1.32) | 1.14  (0.50, 2.61) | Reference | 2.06  (1.48, 2.87) | 8.38  (4.22, 16.63) | >999.999  (<0.001, >999.999) |
| Severe BPD | Reference | 1.57  (0.48, 5.14) | 2.74  (0.78, 9.60) | Reference | 7.52  (1.53, 37.11) | 31.06  (5.13, 187.90) | >999.999  (510.25, >999.999) |
| Discharged with requirement of home oxygen therapy | Reference | 0.91  (0.54, 1.54) | 0.86  (0.29, 2.49) | Reference | 2.32  (1.48, 3.64) | 3.04  (1.41, 6.54) | 23.88  (5.56, 102.62) |
| **26w<GA≤28w** (n=975) |  |  |  |  |  |  |  |
| BPD | Reference | 0.86  (0.57, 1.31) | 0.73  (0.34, 1.56) | Reference | 2.07  (1.43, 3.00) | 7.34  (4.03, 13.39) | 46.07  (13.25, 160.18) |
| Severe BPD | Reference | 0.76  (0.08, 7.00) | 0.99  (0.07, 14.14) | Reference | 3.98  (0.70, 22.52) | 11.07  (1.46, 83.65) | 136.42  (20.15, 923.67) |
| Discharged with requirement of home oxygen therapy | Reference | 1.45  (0.87, 2.40) | 3.79  (1.80, 7.99) | Reference | 1.43  (0.86, 2.39) | 3.06  (1.52, 6.15) | 3.57  (1.45, 8.78) |
| **GA≤26w** (n=305) |  |  |  |  |  |  |  |
| BPD | Reference | 1.59  (0.83, 3.02) | 0.74  (0.28, 1.97) | Reference | 1.58  (0.75, 3.33) | 2.79  (1.20, 6.47) | 13.01  (4.20, 40.33) |
| Severe BPD | Reference | 4.08  (0.17, 95.90) | 11.46  (0.12, >999.999) | Reference | 4.08  (0.24, 70.34) | 1.51  (0.05, 47.61) | 102.93  (4.98, >999.999) |
| Discharged with requirement of home oxygen therapy | Reference | 0.64  (0.28, 1.48) | 0.77  (0.21, 2.81) | Reference | 1.57  (0.48, 5.14) | 2.74  (0.78, 9.60) | 6.17  (1.63, 23.42) |

1. Adjusted for gestational age, birth weight, gender, small for gestational age, 5-min Apgar score, treatment with surfactant, DART treatment, PDA, NEC, sepsis, and mechanical ventilation duration (analyzed as continuous variable).
2. Adjusted for gestational age, birth weight, gender, small for gestational age, 5-min Apgar score, treatment with surfactant, DART treatment, PDA, NEC, sepsis, and mechanical ventilation courses (analyzed as continuous variable).
